# Numerical Simulation of a Core–Shell Polymer Strand in Material Extrusion Additive Manufacturing

**DOI:** 10.3390/polym13030476

**Published:** 2021-02-02

**Authors:** Hamid Narei, Maryam Fatehifar, Ashley Howard Malt, John Bissell, Mohammad Souri, Mohammad Nasr Esfahani, Masoud Jabbari

**Affiliations:** 1 Faculty of New Sciences and Technologies, University of Tehran, Tehran 1439957131, Iran; hamidnarei@ut.ac.ir; 2 Department of Mechanical, Aerospace and Civil Engineering, The University of Manchester, Manchester M13 9PL, UK; maryam.fatehifar@postgrad.manchester.ac.uk; 3 Harvard John A. Paulson School of Engineering and Applied Sciences, Harvard University, Cambridge, MA 02138, USA; aahoward@g.harvard.edu (A.H.M.); msouri@seas.harvard.edu (M.S.); 4 Department of Electronic Engineering, University of York, York YO10 5DD, UK; john.bissell@york.ac.uk (J.B.); mohammad.nasresfahani@york.ac.uk (M.N.E.)

**Keywords:** material extrusion, additive manufacturing, core–shell polymer strand, processing parameters, CFD

## Abstract

Material extrusion additive manufacturing (ME-AM) techniques have been recently introduced for core–shell polymer manufacturing. Using ME-AM for core–shell manufacturing offers improved mechanical properties and dimensional accuracy over conventional 3D-printed polymer. Operating parameters play an important role in forming the overall quality of the 3D-printed manufactured products. Here we use numerical simulations within the framework of computation fluid dynamics (CFD) to identify the best combination of operating parameters for the 3D printing of a core–shell polymer strand. The objectives of these CFD simulations are to find strands with an ultimate volume fraction of core polymer. At the same time, complete encapsulations are obtained for the core polymer inside the shell one. In this model, the deposition flow is controlled by three dimensionless parameters: (i) the diameter ratio of core material to the nozzle, d/D; (ii) the normalised gap between the extruder and the build plate, t/D; (iii) the velocity ratio of the moving build plate to the average velocity inside the nozzle, V/U. Numerical results of the deposited strands’ cross-sections demonstrate the effects of controlling parameters on the encapsulation of the core material inside the shell and the shape and size of the strand. Overall we find that the best operating parameters are a diameter ratio of d/D=0.7, a normalised gap of t/D=1, and a velocity ratio of V/U=1.

## 1. Introduction

Additive manufacturing (AM) has introduced several advantages over conventional methods, such as shortening the design manufacturing cycle, lowering production costs, and increasing the degree of automation [1,2]. Among different technologies, material extrusion AM (ME-AM)—also termed Fused Filament Fabrication (FFF), or Fused Deposition Modelling (FDM)—has been gaining interest. The reason for this attention is its relatively low cost, wide availability, comparatively minor safety concerns regarding the process, and ease of use [3]. In the ME-AM process, 3D parts are formed through the controlled deposition of successive layers of molten material extruded from a moving head along a predefined toolpath [4,5]. Nonetheless, because of the layer-by-layer nature of the deposited material and the existence of numerous voids, parts fabricated by ME-AM suffer from inferior mechanical properties, e.g., low elastic behaviour, possible delamination, and low mechanical integrity [6,7]. Furthermore, layer-based manufacturing methods suffer from rough surfaces, whose post-processing is laborious compared to that of metals [8,9]. Besides, the inherently inferior mechanical properties of filaments, commonly used in ME-AM, exacerbate the position of fabricated parts as fully functional and load-bearing components. This weakness hampers the development of ME-AM from a prototyping role to a process capable of manufacturing finished products [4]. Such imperfections have necessitated the improvement of materials used in ME-AM printed parts to ensure that the structural functionalities of fabricated components comply with the functional requirements of different applications.

**Figure 1:**
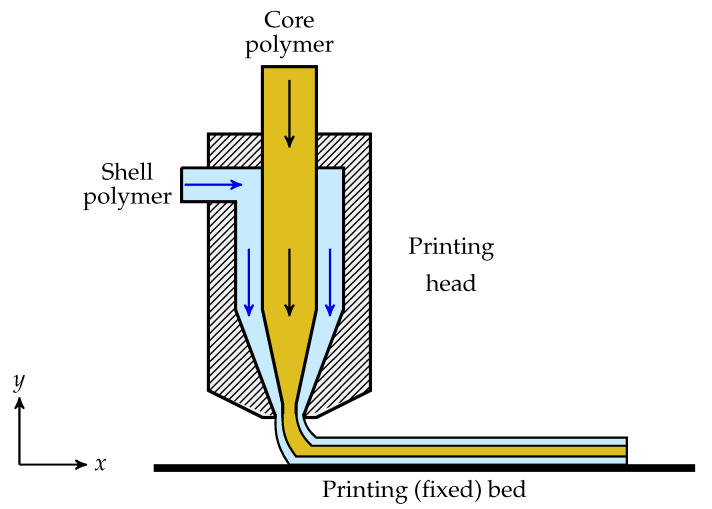
Two-dimensional schematic of core–shell 3D printing.

To overcome some of the mentioned drawbacks, a novel material design approach, namely core–shell structured filament, has recently been developed [10]. In this approach, a polymer resin, favouring from the high glass-transition temperature, such as polycarbonate (PC) [10] or a blend of PC and acrylonitrile-butadiene-styrene (ABS) [11], acts as the core to create a stiff skeleton, reinforcing the printed shape. Another polymer resin with a low glass-transition temperature, such as Surlyn [10] or high/low density polyethylene [11], is used as the shell to enable improved interdiffusion of polymers between adjacent layers. The selected shell polymer should have both crystallinity and ionic functionality to provide routes to enhance the bridging across the interface [10]. In this approach, in order that the core and shell fulfill the role they play in the core–shell structure properly, they should be immiscible to prevent their mixing. In manufacturing samples using the core–shell polymer filament, it has been demonstrated that 3D printed polymeric parts are entitled to unprecedented impact resistance [10], enhanced elongation at break [11] and good dimensional accuracy [10]. In this approach, a two-stage manufacturing process is required to 3D print different products. First, core and shell polymers are melted in two separate extruders at elevated temperature and merge to form the core–shell filament in a coextrusion die. Then, produced core–shell filaments are fed into a heated nozzle in which it is also melted and then deposited onto a build plate through the nozzle [10]. The energy consumed in this process requires two stages of melting of polymers and filaments at elevated temperatures. It may be advantageous for energy reduction to embed the core polymer into the shell inside the printing head and immediately deposit the resulting strand onto the build-plate—c.f. Figure 1. In this approach, only one stage of the melting of polymers is required, and it can also boost the manufacturing speed of parts produced from core–shell structured polymer through 3D printing. Of course, it should be mentioned that such a one-stage process would require a precise control of processing parameters, discussed in the following sections, have the optimal shell thickness, and simultaneously make sure that the core is completely encapsulated inside the shell.

Processing conditions affect the mechanical properties of ME-AM-fabricated parts, fabrication time, and the manufacturing resolution. Strand-to-strand distance plays a crucial role in the mechanical strength, and porosity of the fabricated parts [12,13]. Depending on different parameters (including the volumetric extrusion flux and layer thickness), the bonding area’s width affects the tensile strength, the cross-section, and shape of the deposited strand, and determining it is of paramount importance [8,14]. Furthermore, shape profile—which affects the heat conduction between the adjacent layers and strands—also influences the cooling rate of the fabricated part [8]. The mechanical properties of ME-AM-fabricated parts also depend on the layer thickness, infill density, and build orientation [15]. It is found that surface roughness is strongly correlated with build orientation [16] and layer thickness [17]. To date, studies have focused on optimising the processing parameters of conventional polymers and polymer composites. Thus, further research on the effects of different processing parameters on the performance of the ME-AM-fabricated core–shell structure is of absolute necessity. Since coupling between the parameters makes it difficult to interpret their influence on the component’s performance, the modelling of the process using computational fluid dynamics (CFD) and changing parameters accurately and separately can be extremely useful.

Numerical simulation has been widely employed as a predictive tool to analyse various manufacturing processes [18] and provide a wealth of information about different manufacturing methods, including ME-AM. However, owing to the complex multiphysics phenomena occurring on different temporal and spatial scales [19], the simulation of ME-AM is extremely difficult. Hence, different aspects of the extrusion flow in the ME-AM, including the internal flow of the molten material inside the extruder, the cross-sections of the produced strand immediately after deposition on the build plate, and the thermo-mechanical behaviour of the fabricated parts after the deposition of the strand, are separately simulated by different CFD models. For instance, Xia et al. simulated the temperature distribution within the extruded material in a separate study [20] and the cooling rate, and reheating effect stemming from the deposition of successive layers in another work [21]. Assuming the deposited strand had an elliptic cross-section, McIlroy and Olmsted [22] modelled the deformation and relaxation of the polymer chains of an amorphous polymer melt during the material deposition. Comminal et al. [19] investigated the effects of the layer thickness and the printing speed on the shape of the deposited strand using a 3D CFD model of the strand deposition (assuming a Newtonian fluid); this CFD model was then validated by experiments in [8]. Serdeczny et al. [14] expanded their research further to model the successive deposition of parallel strands to predict the mesostructure formed. They also acquire information about the porosity, the surface roughness, and the inter-and intra-layer bond line densities of the mesostructures. Comminal et al. [5] simulated the material deposition along a toolpath, which included a sharp corner using a CFD model to examine the amount of overfill and underfill in the corners of a toolpath under different operating conditions. In the context of modelling ME-AM printed composites, Heller et al. [23] presented an isothermal CFD model to determine the impacts that extrudate swell and nozzle geometry exert on the fibre orientation. They looked at fibre inside a discrete carbon fibre-reinforced polymeric feedstock. It should be mentioned that they just focused on the feedstock flow inside the nozzle and did not simulate the deposition flow after its exit. There is no study simulating the deposition flow of a core–shell polymer strand using CFD models to the best of the authors’ knowledge.

**Figure 2:**
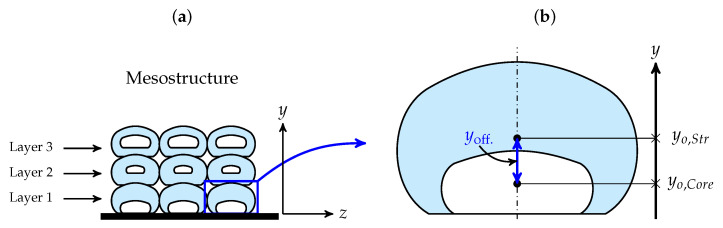
Schematic illustration of the cross-section of (**a**) a 3D-printed mesostructured with three layers, and (**b**) the vertical offset between the centre of the core and the centre of the strand.

In this study, the deposition flow of a core–shell polymer strand is simulated using an isothermal CFD model. Focusing on the deposition flow immediately after the deposition of extruded polymers on the build plate enables us to fully understand the effects of various parameters—including the normalised gap between the extruder and the build plate, the velocity ratio of the moving substrate to the average velocity inside the nozzle, and the diameter ratio of core to the nozzle—on the encapsulation of core inside the shell matrix. As an example, full encapsulation is shown in layers two and three of Figure 2a. In addition to encapsulation, another objective of this study is to obtain strands with the highest volume fraction of core. This may result in better reinforcement of the print shape, thereby improving dimensional accuracy—i.e., layers one and three in Figure 2a. Furthermore, the vertical offset between the centre of the core and the strand centre is investigated in this study—shown in Figure 2b. Ideally, the objective is to produce layers in which each strand has a maximum core that is fully encapsulated and a minimum offset—e.g., layer 3 in Figure 2a.

## 2. Methods

In this article, a core–shell strand extruded from a printing head and deposited in a moving build plate is simulated using a CFD model. The following computation model is adopted to investigate the effects of various parameters on the encapsulation of core into the shell matrix, and the following principles are applied.

### 2.1. Numerical implementation

The material extrusion is generally governed by the Navier—Stokes equations, accounting for mass and momentum conservation. Given that the deposition flow of plastic materials has a deficient Reynolds number (Re<<1) and Froude numbers of around 1 (Fr∼1), hence, the material deposition can be modelled as a creeping flow—similar to [5]. Assumptions are isothermal flow, incompressible flow, Newtonian fluid, creeping flow, and negligible inertial terms. The conservation of mass and momentum becomes:

(1) ∇·u=0

(2) $$\rho \frac{\partial u}{\partial t}=-\nabla p+\mu \nabla^{2}u+\rho g$$

where u is the velocity vector, ρ is the density, *p* is the pressure, μ is the dynamic viscosity, and g is the gravitational body force vector.

The governing equations (i.e., the conservation of mass and momentum) are solved using a coupled pressure–velocity scheme with the implicit time-marching approach and the PRESTO scheme for pressure discretization. Moreover, the Green-Gauss node-based scheme is employed to evaluate gradients, and the QUICK scheme is selected to discretise the convective terms. The volume fractions of the molten polymers and air inside the control volumes are also solved using the implicit modified HRIC scheme [24]. Furthermore, the first order implicit scheme is employed to discretise the transient governing equations of the flow. The free surface of the extruded material and the core and shell polymers interface are tracked using the coupled level-set/volume-of-fluid (VOF) interface capturing method [25,26]. Finally, the time-step increment is set so that the corresponding maximum Courant number is Cr=0.3, in all simulations. The transient simulations are continued until the solutions have reached a steady-state condition. The implementation of the numerical schemes is conducted in ANSYS-Fluent R19.3.

### 2.2. Computational Domain

**Figure 3:**
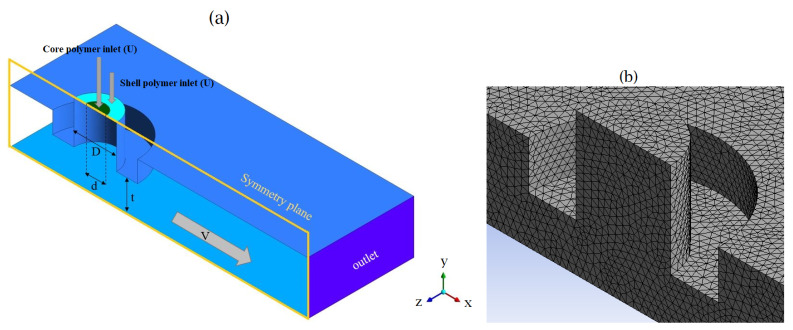
(**a**) Geometry of the computational domain, and (**b**) example of a tetrahedral mesh used in the simulations.

The geometry of the computational model, represented in Figure 3a, comprises the tip of the cylindrical extrusion nozzle and the build volume—that is, the region between the nozzle and build plate. The extrusion nozzle with a diameter of *D* is divided into two parts: the inner one (with a diameter of *d*) for the extrusion of the core polymer and the outer for the shell polymer. The nozzle is positioned over the build plate at the distance of *t*—the so-called extruded layer height. In the simulations, the nozzle head is fixed over the build plate, moving with the constant velocity of *V*. The molten shell and core resins enter the cylindrical nozzle with the same average extrusion volumetric flux of *U*.

The top surface of the nozzle is considered an inlet boundary condition with a fully developed velocity profile. The no-slip boundary condition is applied on all solid walls of the numerical model—i.e., build plate and the nozzle wall—so that the materials fully adhere to them. The numerical model’s remaining external surfaces are prescribed as an outlet boundary condition, where the extruded materials are free to exit the computational domain. Only half of the domain is solved with a symmetry boundary in the xy-plane at z=0. The computational domain is discretised with tetrahedral elements with a maximum normalised edge length of δl/D=0.03—see Figure 3b.

### 2.3. Case Studies

In this study, the extruded material’s deposition flow is simulated for various printing parameters, presented in Table 1. The core polymer’s proper encapsulation into the shell matrix and the maximum volume fraction of the core are the main objectives taken into account here. For these purposes, first, cases with d/D=0.5—whose shell matrix flow rate is the highest among the cases considered in this study—are simulated for other printing parameters, namely t/D and V/U, and the results are then assessed. In the next step, the ratio d/D is increased, and the simulation for other processing parameters is conducted just for the cases where complete encapsulation has occurred for their counterparts with lower d/D. It should be mentioned that increasing the ratio d/D results in a lower flow rate of the shell matrix and a higher core flow rate. Indicating the encapsulation is not possible for the cases with higher d/D whose processing parameters are the same as its counterparts with lower d/D. The gradual increase in the ratio d/D is continued until the cases with different U/V and t/D cannot wholly encapsulate the core.

Assuming the creeping flow, the flow’s viscosity and density do not affect the results of the simulations. However, their value was included in Table 1 for the sake of completeness. As the values of density and viscosity of the surrounding air do not affect the molten polymers’ flow, the air is assigned artificial material properties to facilitate numerical convergence.

Considering the assumptions mentioned above, three dimensionless parameters can entirely determine the deposition flow: the velocity ratio V/U, the normalised layer thickness t/D, and the nozzle diameter ratio d/D. The normalised layer thickness and the nozzle diameter ratio are employed to quantify the computational model’s geometry. In contrast, the velocity ratio is used to parameterise the amount of extruded materials with reference to the printing speed [14]. Given that the numerical results presented in dimensionless forms for fixed values of t/D, d/D, and V/U, the obtained data can be considered equally valid for other simulation cases with various printing speeds nozzle diameters.

## 3. Results and Discussion

**Figure 4:**
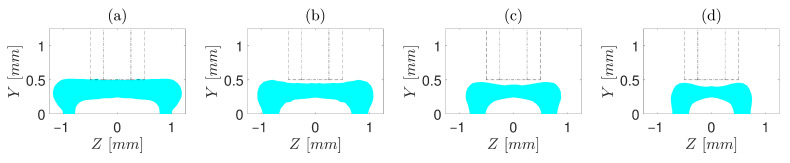
Cross-sections of the core–shell strand for t/D=0.5, d/D=0.5 and different V/U of (**a**) 0.5, (**b**) 0.75, (**c**) 1, and (**d**) 1.25. Core diameter and orifice are marked by “- -” and “-·”, respectively.

**Figure 5:**
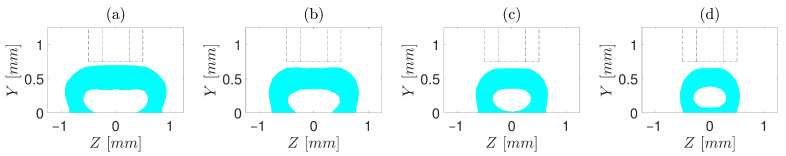
Cross-sections of the core–shell strand for t/D=0.75, d/D=0.5 and different V/U of (**a**) 0.5, (**b**) 0.75, (**c**) 1, and (**d**) 1.25. Core diameter and orifice are marked by “- -” and “-·”, respectively.

**Figure 6:**
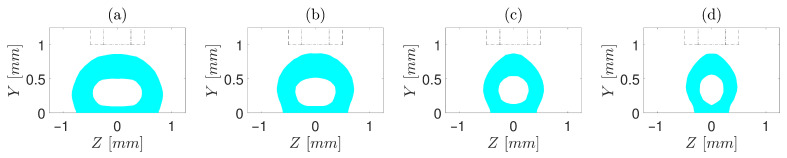
Cross-sections of core–shell strand for t/D=1, d/D=0.5 and different V/U of (**a**) 0.5, (**b**) 0.75, (**c**) 1, and (**d**) 1.25. Core diameter and orifice are marked by “- -” and “-·”, respectively.

The performed simulations include a range of operating conditions, from a slow printing speed with a small gap and a medium flow rate of polymer to a fast printing speed with a large gap and high polymer flow rate. Figure 4, Figure 5 and Figure 6 depict the cross-sections of the simulated core–shell strand for d/D=0.5. Only the shell resin is shown in the cross-sections, and the area between or under the shell resin indicates the core matrix. As demonstrated, the overall cross-sections ranged from being an almost flat cuboid with rounded edges to an egg-shaped cuboid. It is noticed—contrary to common perception—that the vertical gap between the nozzle orifice and the build plate is not exactly equal to the height of the strand, where the height of strand is lower than the gap in most cases—similar to a previous study by Comminal et al. [19]. The difference between the height of the strand and the gap becomes more sensible with increasing t/D or V/U.

Furthermore, comparing cross-sections obtained from operating conditions in which t/D is constant, such as Figure 4a–d, it is clear that increasing V/U reduces the width of the strand. This is mainly stemming from the fact that increasing the volumetric extrusion flux or decreasing the build plate velocity resulted in more accumulation of the extruded material under the nozzle orifice. On the other hand, the comparison between the cross-sections obtained from operating conditions in which V/U is constant, such as Figure 4b, Figure 5b and Figure 6b, demonstrates that the width of the strand also decreases as t/D is increased, mainly due to the radial pressure difference. As the overall shape of the strand deposition flow in ME-AM was previously investigated [19], this study aims not to delve into a more detailed discussion in this regard but to focus more on the encapsulation of the core resin inside the shell after extrusion.

To have a deeper insight, first, cases with d/D=0.5 are investigated (Figure 4, Figure 5 and Figure 6). For t/D=0.5 (Figure 4), regardless of V/U, no encapsulation occurs, which is ascribed to the fact that the nozzle cap forces the strand to be spread over the build plate. In simulations with t/D=0.75 (Figure 5), the encapsulation does not fully form for the slow printing speed (Figure 5a). However, as V/U is increased, it is noticed that the cross-section of the strand moves toward a more favourable shape, such that the full encapsulation is formed for V/U=1 and V/U=1.25 (Figure 5c,d). This is because the higher printing speed (either a lower extrusion volumetric flux or a higher build plate velocity) does not allow the extruded material to stay long enough under the nozzle cap to be spread over the build plate. Eventually, for all cases of t/D=1, the full encapsulation is observed, regardless of V/U (Figure 6a–d). From the point of view of a single strand, all four cases with t/D=1 and d/D=0.5 investigated in this study result in a proper cross-section. Nonetheless, when they are put beside and on top of each other to form a mesostructure, each of these seemingly proper cross-sections may suffer from such deficiencies as high porosity and low inter-and intra-layer bond line densities. Hence, to investigate which of the operating conditions may best serve the mesostructure formed by the successive deposition of strands, simulations of successive strands beside and on top of each other are required, which is beyond this article’s scope.

**Figure 7:**
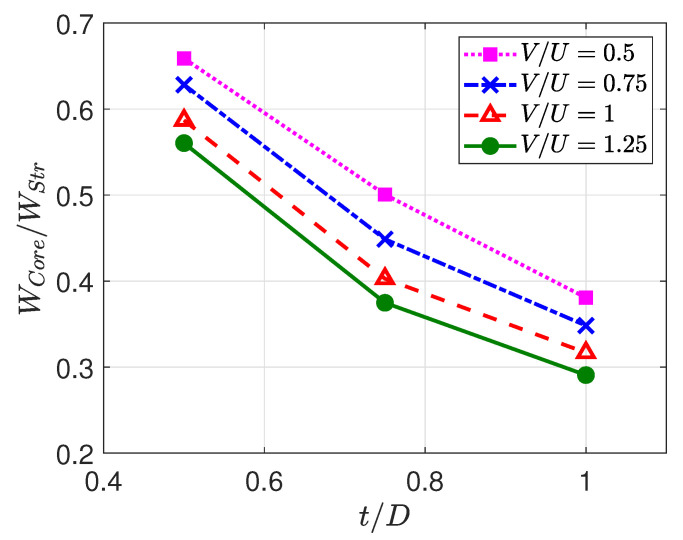
Maximum width of the core to maximum width of the overall strand for different operating parameters as a function of t/D for different V/U.

Before looking into the cases with higher d/D, a discussion about the strand’s width ratio is provided. Figure 7 shows the maximum width of the core ($W_{Core}$) to the maximum width of the strand ($W_{Str}$) for different operating parameters where d/D=0.5. As can be observed, the ratio $W_{Core}/W_{Str}$ is decreased as both V/U and t/D are increased, indicating that the nozzle cap and molten polymer exert lower pressure on the core, and, thereby, the core is spread less evenly over the build plate.

**Figure 8:**
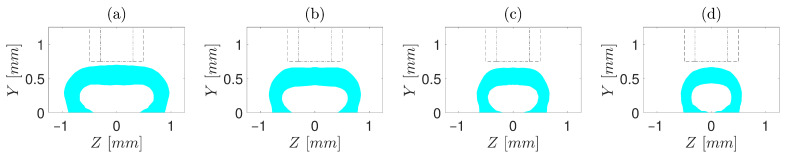
Cross-sections of the core–shell strand for t/D=0.75, d/D=0.6 and different V/U of (**a**) 0.5, (**b**) 0.75, (**c**) 1, and (**d**) 1.25. Core diameter and orifice are marked by “- -” and “-·”, respectively.

**Figure 9:**
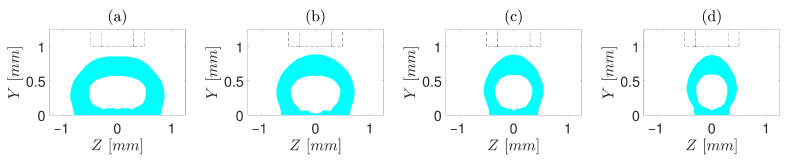
Cross-sections of the core–shell strand for t/D=1, d/D=0.6 and different V/U of (**a**) 0.5, (**b**) 0.75, (**c**) 1, and (**d**) 1.25. Core diameter and orifice are marked by “- -” and “-·”, respectively.

**Figure 10:**
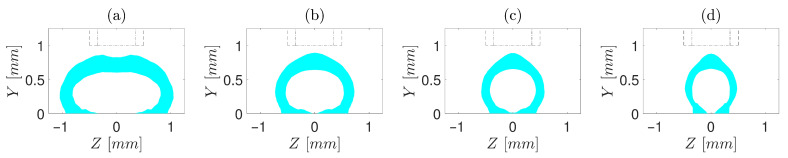
Cross-sections of the core–shell strand for t/D=1, d/D=0.7 and different V/U of (**a**) 0.5, (**b**) 0.75, (**c**) 1, and (**d**) 1.25. Core diameter and orifice are marked by “- -” and “-·”, respectively.

In the next step, cases with d/D=0.6 are simulated and illustrated in Figure 8, Figure 9 and Figure 10. The cross-section of cases with t/D=0.75 is displayed in Figure 8. As seen, encapsulation occurs in none of the investigated cases; hence, from the point of view of encapsulation alone, among the cases with t/D=0.75, the one with t/D=0.5 and V/U=1.25 best fits the objective of this study.

Next, the simulation is performed for cases with d/D=0.6, t/D=1, and various V/U. As depicted in Figure 9, in all cases, the core is fully encapsulated by the shell, indicating that they can be considered proper cases from an encapsulation vantage point. However, to evaluate these cases’ potential from the point of view of the maximum possible volume fraction of core, simulations with higher d/D should be conducted. For this purpose, cases with t/D=1 and d/D=0.7 and different V/U are simulated (Figure 10). It can be observed that the only case in which the shell fully encapsulates the core is V/U=1. Cases with t/D=1 and d/D=0.7 differ somehow from other cases—particularly t/D=0.75 and d/D=0.5. Referring back to Figure 5c,d, as the V/U is increased from 1 to 1.25, better encapsulation was observed. At the same time, for t/D=1 and d/D=0.7, it was shown that the case with V/U=1.25 did not lead to the encapsulation, which could be attributed to a lower volume of molten shell resin existing to cover the entire core.

As the shell resin almost encapsulates the core in the case with t/D=1, d/D=0.7, and V/U=1, it can be concluded that increasing d/D to 0.8 would not result in encapsulation. Hence, this case can be regarded as the most proper instance, simultaneously satisfying both objectives of this study—that is, the maximum possible volume fraction of core to maximize the dimensional accuracy of the resulting strand and the full encapsulation of the core inside the shell to maximize the improvement of interdiffusion of polymers between adjacent layers, resulting in the improved interfaces.

The encapsulation occurred in a case with t/D=1, and d/D=0.7 brings forward another interesting issue, which will be discussed in the following section. First, d/D=0.7 means that the nozzle cap areas where core and shell enter into are almost equal (to be precisely equal, d should be $\sqrt{2}/2≅0.7071$ of *D*). As the velocities of extrusion of core and shell resins are also equal, it can be concluded that the volumetric flow rate is the same and, thereby, the volume of extruded core and shell is almost equal to 50 vol% for each one in d/D=0.7. Second, as discussed above, cases with d/D=0.8 would not result in encapsulation, indicating that the core resin’s higher volume fraction cannot be achieved for these ranges of operating parameters. Hence the maximum volume fraction of the core resin in the core–shell structured strand, with the complete encapsulation of the core inside the shell, is approximately 50 vol%.

**Figure 11:**
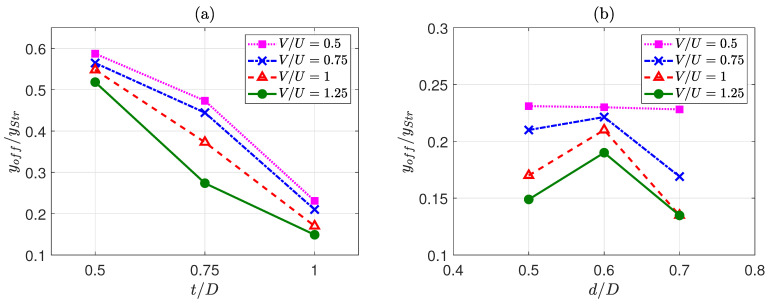
Variation of the normalised offset distance with changing the normalised process parameters when (**a**) d/D=0.5, and (**b**) t/D=1.

Finally, numerical cases are studied in detail from the point of view of the offset between the centres of the core and the overall strand (or the shell centre)—Figure 2b. This is an important point to study, as the increased values of the offset will cause higher anisotropy in the mesostructure and the resultant macro properties. As an example of results, for d/D=0.5, as seen in Figure 11a, the offset ratio generally decreases with the increase in V/U. However, the rate decrease is not the same for different t/D. Cases with t/D=0.5 (no encapsulation occurred for all V/Us) and t/D=1 (full encapsulation occurred for all V/Us) demonstrate a gradual decrease in the offset ratio, while cases with t/D=0.75 experience a sharper decrease after encapsulation occurrence (V/U=1).

In simulated cases with t/D=1—Figure 11b—it is seen that the offset is generally decreased as the V/U is increased. This can be attributed to better encapsulation as the printing speed is increased. Moreover, when d/D is decreased, the offset value is generally increased. However, it is seen that, for d/D=0.5, the trend has changed, leading to a lower amount of the offset. This is due to the fact that at d/D=0.5, the ratio of shell to the core is the highest, and hence there is a better chance of encapsulation. Another interesting data point in Figure 11b is at d/D=0.7 when V=U=1. It can be seen that the offset value has dropped more than expected (compared to other similar situations). The reason behind this change in trend is that this case at V/U=1 experiences full-encapsulation, while the other cases (at d/D=0.7) do not. The occurrence of encapsulation in the case of d/D=0.7 and V/U=1 counters the effect of higher V/U—the case with d/D=0.7 and V/U=1.25. The results presented in Figure 11 confirm that the case with d/D=1, d/D=0.7, and V/U=1 is seen as the best operating point, as this case not only met the two other objectives but now satisfies the third aim (lower value of offset).

## 4. Conclusions

In this study, the deposition flow of an ME-AM-manufactured core–shell structured strand was simulated. Here, a manufacturing process in which the core polymer embedded into the shell inside the heated printing head and the resulting core–shell structure immediately deposited onto the build plate was simulated. The objectives were to find strands with the highest volume fraction of the core to maximize the dimensional accuracy of the resulting strand, the full encapsulation of the core inside the shell resin to maximize the improvement of interdiffusion of polymers between adjacent layers, resulting in the improved interfaces, through altering printing parameters, as well as to minimise the offset between the core centre and the strand centre in order to reduce anisotropy in the structure. Three dimensionless parameters, which included the velocity ratio V/U, the normalised layer thickness t/D, and the nozzle diameter ratio d/D, were used to control the deposition flow of the extruded materials. Using the coupled level-set/volume-of-fluid method, the free surface of the extruded material and the molten core and shell interface were captured, and cross-sections of strands, showing encapsulation, shape, and size, were tracked.

The results showed that increasing d/D will lead to a higher volume fraction of core, and, thus better dimensional accuracy. Moreover, full encapsulations mostly occurred at higher V/U values, which led to lower offset magnitudes. It was demonstrated that the operating parameters of t/D=1, d/D=0.7, and V/U=1 result in a fully encapsulated strand with the highest volume fraction of core, which could be as much as 50 vol%. Furthermore, it was also concluded that the case above shows the least offset magnitude, making the case the best operating data point.

Although some cases offered appropriate cross-sections from the point of view of encapsulation alone, they may suffer from some serious deficiencies, including high porosity and low inter- and intralayer bond line densities, when they are put beside and on top of each other to form a mesostructure. Hence, it is proposed to simulate the successive deposition of strands in order to assess the effect of different operating parameters on the properties of the formed mesostructure for future work. 

## Figures and Tables

**Table 1:** Parameters used in the numerical simulations.

Parameter	Symbol	Value	Unit
Nozzle diameter	*D*	1	mm
Core diameter	*d*	0.5,0.6,0.7	mm
Layer height	*t*	0.5,0.75,1	mm
Average velocity inside the nozzle	*U*	20	mm/s
Build plate velocity	*V*	10,15,20,25	mm/s
Molten shell density	$\rho_{\mathrm{shell}}$	1000	kg/m${}^{3}$
Molten shell viscosity	$\mu_{\mathrm{shell}}$	1000	Pa·s
Molten core density	$\rho_{\mathrm{core}}$	1500	kg/m${}^{3}$
Molten core viscosity	$\mu_{\mathrm{core}}$	1500	Pa·s
Air density	$\rho_{\mathrm{air}}$	0.01	kg/m${}^{3}$
Air viscosity	$\mu_{\mathrm{air}}$	0.01	Pa·s

## Data Availability

Data is contained within the article.

## References

[1] Turner B.N., Gold S.A. (2015). A review of melt extrusion additive manufacturing processes: II. Materials, dimensional accuracy, and surface roughness. Rapid Prototyp. J..

[2] Nath S.D., Nilufar S. (2020). An Overview of Additive Manufacturing of Polymers and Associated Composites. Polymers.

[3] Fallon J.J., McKnight S.H., Bortner M.J. (2019). Highly Loaded Fiber Filled Polymers for Material Extrusion: A Review of Current Understanding. Addit. Manuf..

[4] Chacón J., Caminero M., Núñez P., García-Plaza E., García-Moreno I., Reverte J. (2019). Additive manufacturing of continuous fibre reinforced thermoplastic composites using fused deposition modelling: Effect of process parameters on mechanical properties. Compos. Sci. Technol..

[5] Comminal R., Serdeczny M.P., Pedersen D.B., Spangenberg J. (2019). Motion planning and numerical simulation of material deposition at corners in extrusion additive manufacturing. Addit. Manuf..

[6] Araya-Calvo M., López-Gómez I., Chamberlain-Simon N., León-Salazar J.L., Guillén-Girón T., Corrales-Cordero J.S., Sánchez-Brenes O. (2018). Evaluation of compressive and flexural properties of continuous fiber fabrication additive manufacturing technology. Addit. Manuf..

[7] Blok L.G., Longana M.L., Yu H., Woods B.K. (2018). An investigation into 3D printing of fibre reinforced thermoplastic composites. Addit. Manuf..

[8] Serdeczny M.P., Comminal R., Pedersen D.B., Spangenberg J. (2018). Experimental validation of a numerical model for the strand shape in material extrusion additive manufacturing. Addit. Manuf..

[9] Verbeeten W.M.H., Arnold-Bik R.J., Lorenzo-Bañuelos M. (2021). Print Velocity Effects on Strain-Rate Sensitivity of Acrylonitrile-Butadiene-Styrene Using Material Extrusion Additive Manufacturing. Polymers.

[10] Peng F., Zhao Z., Xia X., Cakmak M., Vogt B.D. (2018). Enhanced Impact Resistance of Three-Dimensional-Printed Parts with Structured Filaments. ACS Appl. Polym. Mater..

[11] Peng F., Jiang H., Woods A., Joo P., Amis E.J., Zacharia N.S., Vogt B.D. (2019). 3D Printing with Core–Shell Filaments Containing High or Low Density Polyethylene Shells. ACS Appl. Polym. Mater..

[12] Serdeczny M.P., Comminal R., Pedersen D.B., Spangenberg J. (2018). Numerical prediction of the porosity of parts fabricated with fused deposition modeling. Proceedings of the 29th Annual International Solid Freeform Fabrication Symposium (SFF Symp 2018).

[13] Bakradze G., Arājs E., Gaidukovs S., Thakur V.K. (2020). On the Heuristic Procedure to Determine Processing Parameters in Additive Manufacturing Based on Materials Extrusion. Polymers.

[14] Serdeczny M.P., Comminal R., Pedersen D.B., Spangenberg J. (2019). Numerical simulations of the mesostructure formation in material extrusion additive manufacturing. Addit. Manuf..

[15] Li H., Wang T., Yu Z. (2017). The quantitative research of interaction between key parameters and the effects on mechanical property in FDM. Adv. Mater. Sci. Eng..

[16] Thrimurthulu K., Pandey P.M., Reddy N.V. (2004). Optimum part deposition orientation in fused deposition modeling. Int. J. Mach. Tools Manuf..

[17] Anitha R., Arunachalam S., Radhakrishnan P. (2001). Critical parameters influencing the quality of prototypes in fused deposition modelling. J. Mater. Process. Technol..

[18] Jabbari M., Baran I., Mohanty S., Comminal R., Sonne M.R., Nielsen M.W., Spangenberg J., Hattel J.H. (2018). Multiphysics modelling of manufacturing processes: A review. Adv. Mech. Eng..

[19] Comminal R., Serdeczny M.P., Pedersen D.B., Spangenberg J. (2018). Numerical modeling of the strand deposition flow in extrusion-based additive manufacturing. Addit. Manuf..

[20] Xia H., Lu J., Dabiri S., Tryggvason G. (2018). Fully resolved numerical simulations of fused deposition modeling. Part I: Fluid flow. Rapid Prototyp. J..

[21] Xia H., Lu J., Tryggvason G. (2018). Fully resolved numerical simulations of fused deposition modeling. Part II–solidification, residual stresses and modeling of the nozzle. Rapid Prototyp. J..

[22] McIlroy C., Olmsted P.D. (2017). Deformation of an amorphous polymer during the fused-filament-fabrication method for additive manufacturing. J. Rheol..

[23] Heller B.P., Smith D.E., Jack D.A. (2016). Effects of extrudate swell and nozzle geometry on fiber orientation in Fused Filament Fabrication nozzle flow. Addit. Manuf..

[24] Jabbari M., Bulatova R., Hattel J.H., Bahl C.R. (2014). An evaluation of interface capturing methods in a VOF based model for multiphase flow of a non-Newtonian ceramic in tape casting. Appl. Math. Model..

[25] Hirt C.W., Nichols B.D. (1981). Volume of fluid (VOF) method for the dynamics of free boundaries. J. Comput. Phys..

[26] Sethian J.A., Smereka P. (2003). Level set methods for fluid interfaces. Annu. Rev. Fluid Mech..

